# Prevalence of dental caries and associated risk factors in children living with disabilities in Rwanda: a cross-sectional study

**DOI:** 10.11604/pamj.2020.36.193.24166

**Published:** 2020-07-17

**Authors:** Donat Uwayezu, Agnes Gatarayiha, Manassé Nzayirambaho

**Affiliations:** 1 School of Dentistry, College of Medicine and Health Sciences, University of Rwanda, Kigali, Rwanda,; 2 School of Public Health, College of Medicine and Health Sciences, University of Rwanda, Kigali, Rwanda

**Keywords:** Dental caries, child, disability, Rwanda

## Abstract

**Introduction:**

several studies have been done on children with disabilities, and the results have shown that these particular individuals are more prone to developing various abnormal oral conditions. However, little is known about the oral health conditions among children with disabilities in Rwanda. This study aims to determine the prevalence of dental caries and associated risk factors among children with disabilities.

**Methods:**

a cross-sectional study conducted among 226 randomly selected children living with physical disabilities; learning, intellectual and developmental disabilities; deafness, blindness and hearing impairment disabilities aged between 7 and 20 years old, who live and/or are under the care of NYANZA Home de la Vierge des Pauvres (HVP) GATAGARA. Bivariate and logistic regression analyses were done using SPPS version 20 at 95% confidence interval. The significance level was set at p<0.05.

**Results:**

the prevalence of dental caries found in children with disabilities is 42.4%. In bivariate analysis age (p=0.003), frequency of sugary food consumption (p=0.001) and oral hygiene status (p=0.000) are respectively significantly associated with dental caries. In logistic regression model, children who take once or more times per day sugary food like biscuits, cake, chocolates and sweets are almost 6 times higher at risk of developing dental caries [OR: 5.945, CI: 1.187; 29.774, P=0.03) while a good oral hygiene status was protective against dental caries [OR: 0.296, CI: 0.159; 0.550, P=0.000].

**Conclusion:**

dental caries is a reality among children living with disabilities. Appropriate measures should be taken to protect these children and these measures should mainly focus on identified factors.

## Introduction

Dental caries is the most widespread chronic disease with negative impact to the global public health by destroying health teeth. Worldwide, different findings have shown that most children and an estimated 90% of adults have experienced caries, with the disease most prevalent in the Middle East, Latin America and south Asia [1,2]. Different studies have shown that the prevalence of dental caries in children with disabilities is high compared to other general population. This is due to the fact that the level of self care on oral health in children with disabilities is low due to inadequate support from their parents and guardians and inability to communicate their oral health needs due to mental or physical disabilities they have [3-5]. In developed countries dental caries prevalence is still high especially in children with disabilities. United States surgeon general´s report have shown that 45% of children aged between5 and 17 years old have dental caries and the severity of the problem increases in children with disabilities [6]. This is similar to the study done in Turkey also showed a high frequency of dental caries and poor oral hygiene among people attending special schools of disabilities where 86.4% among the participants had dental caries. One in three subjects examined had heavy plaque accumulation which may increase the risk of dental caries development among these children [7].

This problem of dental caries in children with disabilities is not only high in developed countries. Also the burden of the disease in some middle income countries is high where like in India, among special schools of disabled children located in the State of Delhi, 38% of the children with disabilities have been shown to have dental caries and among them 55% suffer from gingival bleeding due to poor oral hygiene [8]. All these problems are marked either because of their actual disability, or for other medical condition like taking medicines with high sugary content or excessive tooth grinding with self-mutilating behaviors or social-economic reason and parents who are not able to carry out the proper oral hygiene of them [6-8]. In Africa, dental caries is not considered among the more serious diseases whereas at individual level, it causes a great suffering, pain and burden. The prevalence of the disease varies greatly between and within countries as well as within different groups of the population [9]. One of these groups affected by dental caries is a group of children living with disabilities whereas a study done in South Africa in 2012 about prevalence of dental caries among children attending special needs schools in Johannesburg showed a prevalence of 28 and 33% dental caries in primary and permanent teeth among children with disability [10]. Although many studies in different countries have found out the impact of dental caries to oral health and general health of children living with disabilities [4,5,11,12], little or no information have been shown in Rwanda to determine the prevalence of dental caries and associated risk factors in children with disabilities. This might be explained by limited accessibility of professional oral services and personal oral care especially to this group of people, hence, there is a need of advocacy to provide possible preventive and treatment interventions based on the results from this study. This study aimed to determine the prevalence of dental caries and associated risk factors involved in this caries development among the children living with Disabilities.

## Methods

**Study design, setting and population:** this study was a quantitative cross-sectional design conducted at NYANZA Home de la Vierge des Pauvres (HVP) GATAGARA. The study population was all children aged between 7and 20 years old living with physical disabilities; learning, intellectual and developmental disabilities; deafness, blindness and hearing impairment disabilities both males and females of NYANZA HVP GATAGARA. Among these children, some live in the Center and others live out of Center with their parents but followed by HVP GATAGARA. Home de la Vierge des Pauvres (HVP) Gatagara is the institution working in favour of persons with disability. It started in 1962 and it was the first and only one center for medical care, education and reintegration of people with disability in Rwanda. Its vision is to radiate God's love to People with Disability through healthcare and special education and mission of giving High quality and sustainable education, orthopedic and rehabilitation services to all persons with physical disabilities in partnership with other stakeholders. In Rwanda, there are few known special centers which take care of children with special needs include NYANZA HVP GATAGARA (multiple disabilities Center), GIKONDO HVP GATAGARA (mental disabilities Center) and RWAMAGANA HVP GATAGARA (blindness disabilities Center). However, NYANZA HVP GATAGARA was chosen as a Center of different multiple disabled children.

**Sample size and sampling techniques:** a simple random sampling method was used to select study participants. The overall sample size was 227 participants.

**Inclusion criteria:** the children with physical disabilities; learning, intellectual and developmental disabilities; deafness, blindness and hearing impairment disabilities whose parents or guardians consented to participate in the study. Children with disabilities unable to assent and answer questions also were included in the study and checked for examination and their parents or guardians answered the questionnaires in their place.

**Exclusion criteria:** children with parental consent or guardians consent but who were unable to cooperate during the clinical oral examination were excluded from the study. Children who were not available on the days of data collection and Children with no parental or guardians consent were also excluded.

**Data collection method and procedures:** in the process of data collection, firstly researchers gave interviews to participants by answering questions of questionnaire which was formulated and translated in Kinyarwanda (local language). Thereafter, children were examined to assess dental caries and oral hygiene status. This procedure of examination was done by a team of volunteer dental therapists who were trained about this study. Caregivers helped to identify each type of disability. A structured questionnaire used was consisted of 3 sections: the first section was concerned with social-demographic factors and type of disability; the second section was concerned with questions on oral health behavior factors and section three was assessment of dental caries and oral hygiene status. This questionnaire was a modified version of a questionnaire utilized in a previous study for disabled children to assess factors associated with their oral health status [13]. The questionnaire was pre-tested in 30 children living with the disabilities not participating in this study. Clinical oral examination was done using tongue depressor and a mouth mirror to assess caries experience by looking the presence or absence of existing caries and oral hygiene status by looking the presence or absence of dental plaque and calculus. The examination for those children was done at their school under natural light. Assessment of oral hygiene status criteria was calculated using the Simplified Oral Hygiene Index of Greene and Vermillion. The oral hygiene of each child was classified as ‘good’ when 0-3 sextants have dental plaque and/or calculus visible, and ‘poor’ when 4-6 sextants have dental plaque with/or calculus visible [14].

**Data analysis:** data was entered into Statistical Package for Social Sciences (SPSS) version 20. Association between demographic factors, oral health behaviors factors and Dental caries was studied using bivariate analysis. Variables that showed significant associations with dental caries were entered in logistic regression model to identify their relative importance in explaining the occurrence of dental caries. The significance level was set at p < 0.05.

**Ethical considerations:** the study material was reviewed and approved by the Institutional Health Research Ethics Review Committee of the College of Medicine and Health Sciences Sciences of University of Rwanda. Permission was obtained from the directorate of NYANZA HVP GATAGARA. Written consent was obtained from parents or guardians of the children before participating in the study. Only children who who had a willing to participate were included in the study and signed assent form.

## Results

**Social-demographic characteristics of the participants:** a total of 226 disabled children participated in the study with 100% response rate. From the participants 123(54.4%) were females and 103(45.6%) were males. The majority of children, 117(51.8%), were in the age group of 13-20 years old. Among them, 81(35.8%) were in the category of physical disabled children, 59(26.1%) were in the category of developmental, intellectual and learning disabled children whereas 86(38.1) were in the category of deaf, blind and hearing impaired children (Table 1).

**Table 1:** social demographic characteristics of the children living with disabilities at HVP Gatagara, Rwanda

Variables	Frequency	(%)
**Age(years)**		
7-12yrs	109	(48.2)
13-20yrs	117	(51.8)
**Gender**		
Male	103	(45.6)
Female	123	(54.4)
**Types of disability**		
Physical disability	81	(35.8)
Developmental, intellectual and learning disability	59	(26.1)
Others (deaf, blind, hearing impairment etc...)	86	(38.1)
**Living location**		
Within the school	129	(57.1)
Outside the school	97	(42.9)

**Prevalence of dental caries among the participants:** in general, 42.4% of the participants had dental caries. The prevalence in males was 42.7% while in female was 42%. With regard to the age, the highest prevalence was in the age group of 7 to 12 years old (47.7%). Considering types of disability the high prevalence of dental caries was found in children with developmental, intellectual and learning disabilities (44%) followed with children with physical disabilities (42%) while in the other types of disabilities such as deaf, blind, hearing impairment was 41.9%. Among all these children, those who live outside the school with their parents also found to have high prevalence of dental caries (44.3%) compared to those who live within the school (41%). children who take once or more times sugary food consumption per day had a high prevalence of developing dental caries disease (59.7%) compared to those who take them few times or never (36.6%). Also a high prevalence of dental caries was found in children who do not use fluoride toothpaste (62.5%) during teeth brushing and those with poor oral hygiene (62.6%) (Table 2).

**Table 2:** prevalence of dental caries among the children living with disabilities at HVP Gatagara, Rwanda

Variables	Dental caries		P-Value
	YES N (%)	NO N (%)	
**Total Prevalence**	**96(42.4)**	**130(57.6)**	
**Age(years)**			**0.003**
7-12yrs	57(47.7)	52(52.3)	
13-20yrs	39(35.7)	80(64.3)	
**Gender**			**0.527**
Male	44(42.7)	59(57.3)	
Female	52(42.2)	71(57.7)	
**Types of disability**			**0.959**
Physical disability	34(42)	47(58)	
Developmental, Intellectual and learning disability	26(44)	33(56)	
Others (deaf, blind, hearing impairment etc...)	36(41.9)	50(58.1)	
**Living location**			**0.362**
Within the school	53(41.0)	76(59.0)	
Outside the school	43(44.3)	43(44.3)	
**Frequency of sugary food consumption**			**0 .001**
Once or more times per day	34(59.6)	23 (40.4)	
Few times per week or never	62(36.6)	107(63.3)	
**Use of tooth paste**			**0.210**
Yes	91(41.7)	127(58.3)	
No	5(62.5)	3(37.5)	
**Oral hygiene status**			**0.000**
Good	44(30.7)	99(69.3)	
Poor	52(69.3)	21(37.4)	

**Factors associated with dental caries:** age, frequency of sugary food consumption and oral hygiene status were respectively statistical significantly associated with dental caries among children living with disabilities (p=0.003, p=0.001, p=0.000) while gender, types of disabilities, living location and use of fluoride toothpaste were not statistically significantly associated with dental caries among children living with disabilities (Table 2). According to results those who take once or more times per day sugary food like biscuits, cake, chocolates and sweets are almost 6 times higher at risk of developing dental caries [OR: 5.945, CI: 1.187; 29.774, P=0.03] while a good oral hygiene is protective against dental caries [OR: 0.296, CI: 0.159; 0.550, P=.000] as shown in (Table 3).

**Table 3:** factors associated with dental caries in children with disabilities at HVP Gatagara, Rwanda

Variable	OR	95% CI	P-Value
**Age**			
7-12years	2.425	[0.996; 5.904]	**0.051**
13-20years	1.051	[0.451; 2.447]	0.909
**Frequency of sugary food consumption**			
Once or more times per day	5.945	[1.187;29.774]	**0.030**
Once week or few times or never	0.934	[0.288;3.032]	0.910
**Oral health status**			
Good(0-3 sites with plaque and/or calculus visible)	0.296	[0.159;0.550]	**0.000**

## Discussion

In this study, dental caries prevalence among children living with disabilities was found to be 42.4% with no significant difference between types of disabilities. This prevalence is high compared other African countries like South-Africa (27.55%), Nigeria (33.3%), and Tanzania (23%) [10,15,16]. This difference could be caused by the fact that in Rwanda there is no oral health guidelines specifically designed to help children living with disabilities to maintain good oral health and the accessibility to oral health services is limited. It may also be associated with lack of people specialized in oral health care for children with disabilities. Our prevalence however is low compared to what other many studies found in middle and developed countries like China (53,5%), India (60%), Kuwait (88.8%), South-Arabia (88,2%), Turkey (84.6%), Bulgaria (89%) [5,7,17-19]. This difference could be caused by that generally children in developed countries are more exposed to sugary food consumption compared to African children as one of the factors contributing to the development of dental caries [20].

In this current study, age of participants found to be associated with dental caries prevalence where in the group age of 7 to 9 years old had a high prevalence (63.9%) compared to other aged groups. These findings are similar to the results reported by other researchers [21,22] who showed that age is one of the factors which are associated with dental caries in children with disabilities. The high prevalence in this category of the age compared to others could be explained that children at this age category, their capacity of taking good oral care through proper might be low compared to others age category. This may influence increase in caries prevalence compared to others with above ages. As revealed to the results, also frequency of sugary food consumption like biscuits, cake, chocolates and sweets found to be associated with dental caries in children with disabilities. These results are similar to the results done in other many studies [23-29] showed that the frequency of sugary food consumption influences an occurrence of dental caries. This is explained by the factor that sugary food in the mouth is changed into acid by cariogenic bacteria such us *Streptococcus mutans* and lactobacilli and that acid start demineralization of tooth and cause dental caries disease on that tooth.

In addition, oral hygiene status was also significant associated with dental caries in children with disabilities where children with poor oral hygiene were 6 times of developing dental caries compared to children with good hygiene. This finding is consistent with other studies [30-33]. This is due to the that children with poor oral hygiene had dental plaques which normally contain *Streptococcus mutans* and lactobacillus types of bacteria that involved in initiation and development of dental caries. Limitation in mental and physical abilities of these children with disabilities may resulted in difficulty in tooth brushing, as well as limited understanding of the importance of oral hygiene care and difficulty of the participants in communicating oral health needs together with their fear of oral health procedures which may have influence in development of dental caries diseases [7]. Gender, living location, types of disability of the participants and tooth paste use were not significantly associated with occurrence of dental caries in these children living with disabilities. Given the context, the prevalence of dental caries is high among children living with disabilities and this is a public health problem concern where different agencies and institutions should focus on. Therefore, oral health education and other preventives measures should be taken to help these children living with disabilities mainly by focusing on the risk factors found to be associated with this disease in children with disabilities.

**Limitation of the study:** these findings are not generalizable to the whole community of children with disabilities in Rwanda because the study was conducted at NYANZA HVP GATAGARA, one of the special centers of children living with disabilities in Rwanda.

## Conclusion

The findings show that dental caries are reality and prevalent among children living with disabilities in Rwanda. The study identified risk factors significantly associated with dental caries are oral hygiene status, frequency of sugary food consumption and age. Therefore appropriate intervention measures should be taken in order to protect the children living with disabilities and these measures should mainly focus on identified factors.
